# Annotation-efficient deep learning detection and measurement of mediastinal lymph nodes in CT

**DOI:** 10.1007/s11548-025-03513-y

**Published:** 2025-09-13

**Authors:** Alon Olesinski, Richard Lederman, Yusef Azraq, Jacob Sosna, Leo Joskowicz

**Affiliations:** 1 School of Computer Science and Engineering, The Hebrew University of Jerusalem, Jerusalem, Israel; 2 Department of Radiology, Hadassah University Medical Center, Jerusalem, Israel

**Keywords:** Semi-supervised deep learning, Lymph node detection and segmentation, Annotation efficiency, Observer variability

## Abstract

**Purpose:**

Manual detection and measurement of structures in volumetric scans is routine in clinical practice but is time-consuming and subject to observer variability. Automatic deep learning-based solutions are effective but require a large dataset of manual annotations by experts. We present a novel annotation-efficient semi-supervised deep learning method for automatic detection, segmentation, and measurement of the short axis length (SAL) of mediastinal lymph nodes (LNs) in contrast-enhanced CT (ceCT) scans.

**Methods:**

Our semi-supervised method combines the precision of expert annotations with the quantity advantages of pseudolabeled data. It uses an ensemble of 3D nnU-Net models trained on a few expert-annotated scans to generate pseudolabels on a large dataset of unannotated scans. The pseudolabels are then filtered to remove false positive LNs by excluding LNs outside the mediastinum and LNs overlapping with other anatomical structures. Finally, a single 3D nnU-Net model is trained using the filtered pseudo-labels. Our method optimizes the ratio of annotated/non-annotated dataset sizes to achieve the desired performance, thus reducing manual annotation effort.

**Results:**

Experimental studies on three chest ceCT datasets with a total of 268 annotated scans (1817 LNs), of which 134 scans were used for testing and the remaining for ensemble training in batches of 17, 34, 67, and 134 scans, as well as 710 unannotated scans, show that the semi-supervised models’ recall improvements were 11–24% (0.72–0.87) while maintaining comparable precision levels. The best model achieved mean SAL differences of 1.65 ± 0.92 mm for normal LNs and 4.25 ± 4.98 mm for enlarged LNs, both within the observer variability.

**Conclusion:**

Our semi-supervised method requires one-fourth to one-eighth less annotations to achieve a performance to supervised models trained on the same dataset for the automatic measurement of mediastinal LNs in chest ceCT. Using pseudolabels with anatomical filtering may be effective to overcome the challenges of the development of AI-based solutions in radiology.

**Supplementary Information:**

The online version contains supplementary material available at 10.1007/s11548-025-03513-y.

## Introduction

Manual detection and measurement of structures of interest in volumetric scans is routine in clinical practice. However, these tasks are time-consuming and subject to observer variability. In recent years, a variety of automatic volumetric image analysis methods have been developed to address these needs.

Supervised deep learning models, e.g., the U-Net [1] and its successors, nnU-Net [2], have become the method of choice for the automatic detection and segmentation of structures in medical images based on voxel-level classification [3]. For example, the nnU-Net-based TotalSegmentator [4] segments 104 anatomical structures (27 organs, 59 bones, 10 muscles, and eight vessels) in CT scans with a mean Dice score of 0.94. Training these models, however, requires a large set of expert-annotated CT scans, which is often unavailable or difficult to obtain.

To address the unmet need of image-based oncology staging, treatment planning, and outcome prediction, we have developed a fully automatic end-to-end pipeline, called SimU-Net, for multi-organ, multi-modality comprehensive detection and segmentation of cancer lesions and the analysis of their evolution over time in longitudinal studies [5–8]. The SimU-Net pipeline combines model-based and fully supervised deep learning modules trained and tested with thousands of manual expert-annotated lesion delineations.

To expand the scope of the analysis, the identification of enlarged lymph nodes is required [9]. The guidelines require measurement of enlarged lymph nodes whose short axis length (SAL) is > 10 mm [10, 11]. Manual detection and measurement of lymph nodes is time-consuming and subject to observer variability: radiologists have to locate the lymph nodes in the CT slices and measure those that are suspected to be enlarged. With tens of lymph nodes in various locations, some appearing in clusters and with fuzzy boundaries, it may lead to missed lymph nodes and inaccurate measurements. Thus, developing methods for lymph node detection and measurement is required. However, producing manual annotations of lymph nodes needed to train fully supervised models is impractical.

Semi-supervised methods aim to reduce the annotation burden by using annotated and unannotated data [12, 13]. Teacher-student methods employ dual networks that use computed pseudolabels of unannotated data for supervised training [14]. Other methods incorporate uncertainty estimation to improve the reliability of computed pseudolabels [15, 16]. While these methods are promising, they perform poorly on small structures, are hard to adapt to new structures, and are computationally expensive.

Recent deep learning approaches for lymph node analysis have been developed for various anatomical regions and imaging modalities [17, 18]. Oda et al. [19] use a 3D U-Net with auxiliary anatomical labels of lungs, airways, aortic arches, and pulmonary arteries. They report a 0.95 recall with 16.3 false positives/scan and a Dice score of 0.52 for lymph nodes > 5 mm. Bouget et al. [20] describe a pipeline that combines a Mask R-CNN for mediastinal lymph node detection and 2D U-Net to segment 15 anatomical structures. They report a 0.75 recall with 9 false positives/scan. Mathai et al. [21] use an ensemble of 3D nnU-Net models jointly trained on labels of lymph nodes and of 28 anatomical structures computed with TotalSegmentator. They report a precision of 0.92, recall of 0.64, and Dice score of 0.68 for lymph nodes > 8 mm. None of these methods is sufficiently accurate and reliable for clinical use.

Several works have studied the observer variability in lymph node detection and measurements on CT [22, 23]. They report a wide range of variability of 41–94% depending on the nodal stations, imaging protocol, expertise, and lymph node size. McErlean et al. [24] report a detection agreement of 94.5% for lymph nodes > 10 mm and a SAL measurement variability of − 11.6% to 6.7% (17 radiologists, 320 CT scans). Hopper et al. [25] report SAL measurement observer variability of 3–15%, with an additional 5% variability for poorly defined or irregularly shaped lymph nodes. Fabel et al. [26] report a mean absolute difference of 3.9% and 13.8% between manual and computed measurements on CT scans with 1.5-mm and 5-mm slice thickness, respectively (85 lymph nodes).

We present a novel annotation-efficient deep learning method for automatic detection, segmentation, and measurement of the SAL of mediastinal lymph nodes in contrast-enhanced CT (ceCT) scans. Our semi-supervised approach is unique in that it combines the precision of expert annotations with the quantitative advantages of pseudolabeled data, while incorporating anatomical context through structure-based filtering. Our method requires one-fourth to one-eighth less annotated data to achieve a performance that is similar to a fully supervised method.

The contributions of this paper are: (1) a semi-supervised learning approach that combines expert annotations with pseudolabels for mediastinal lymph node detection, segmentation, and SAL measurement; (2) an anatomical filtering strategy that reduces false positive detections using anatomical constraints derived from mediastinal structure segmentations; and (3) experimental results that quantify the performance of the method and the observer variability.

## Method

**Fig. 1:**
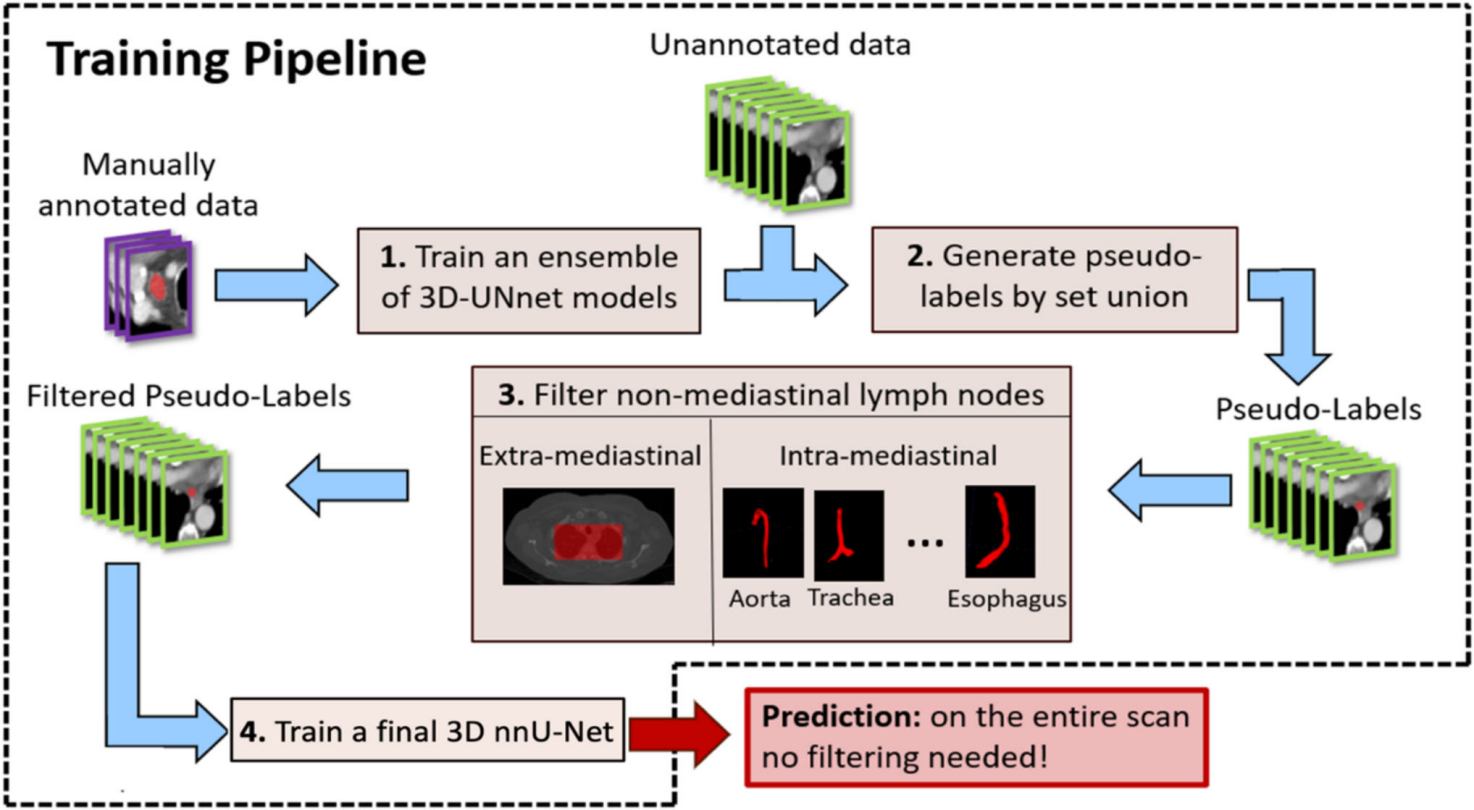
Overview of the annotation-efficient training pipeline for mediastinal lymph node detection and segmentation: (1) training of an ensemble of 3D nnU-Net models on a small set of expert-annotated scans; (2) generation of pseudolabels on unannotated scans through voxel-wise union of ensemble labels; (3) filtering of pseudolabels using anatomical constraints to remove non-mediastinal computed lymph nodes and those overlapping with key structures; and (4) training of a final 3D nnU-Net on the filtered pseudolabels for inference on new CT scans. The resulting 3D nnU-Net is the one used for inferencing as is, with no filtering or pseudolabel generation

The method is an annotation-efficient training pipeline for automatic detection and segmentation of mediastinal lymph nodes in ceCT scans using a novel semi-supervised deep learning approach (Fig. 1). It consists of four steps: (1) ensemble training: train an ensemble of 3D nnU-Net models on a few expert-annotated CT scans; (2) pseudolabel generation: generate initial lymph node segmentations on unannotated scans by combining computed labels from the ensemble models; (3) anatomical filtering: remove false positive lymph nodes using anatomical constraints of mediastinal structures; and (4) final model training: train a single 3D nnU-Net model using the filtered pseudolabels.

*Step 1: Ensemble training:* The first step creates multiple models trained on the few annotated datasets using ensemble learning [27]. They are 3D nnU-Net models trained with a loss function which is the sum of the Dice and the cross-entropy losses. The training of 24 models is performed with a cyclical learning rate schedule in each training run consisting of six cycles of 1000 epochs each. Within each cycle, the learning rate starts at 0.1 and gradually decreases until epoch 900 and remains constant at 0.01 for the final 100 epochs. This constant learning rate phase prevents the model from converging to a single solution, enabling the generation of different and equally plausible models. For each of the six cycles, the last four model checkpoints from the constant learning rate phase are retained, yielding a total of 24 models. The high initial learning rate at the start of each new cycle helps ensure diversity between cycles, causing the optimization to converge toward different solutions.

*Step 2: Pseudolabels generation:* The 24 models are run on the unlabeled datasets, generating voxel-level pseudolabels, which are then combined with a voxel-wise union operation. Thus, voxels for which at least one of the models labeled it as being part of a lymph node are included in the final labeling. This union strategy was chosen over more conservative ones, e.g., majority voting or thresholding, since it prioritizes a low false negative rate, thus minimizing missed lymph nodes, which are later irrecoverable. While this increases false positives, those are filtered out in the next step. Lymph node segmentations are obtained by computing 3D connected components and applying morphological operations to fill-in holes and remove small components ($\le$ 30 voxels, ~ 3 mm).

**Fig. 2:**
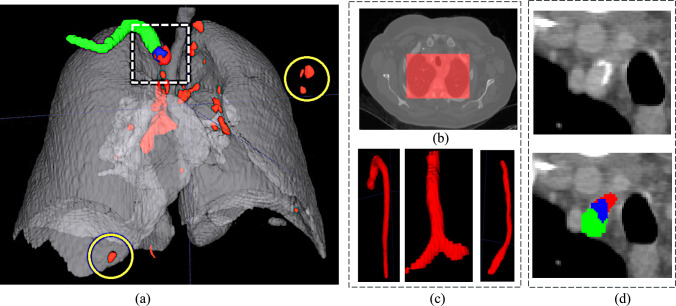
Illustration of anatomical filtering to remove computed false positive lymph nodes (Step 3): **a** computed lymph nodes (red) inside the lungs (gray); **b** extramediastinal filtering removes lymph nodes outside the mediastinal region (dotted white box) – the yellow circles show two excluded examples; **c** intramediastinal filtering removes computed lymph nodes inside the mediastinum based on anatomical part segmentations; **d** part of a lymph node (blue) overlaps with the right subclavian artery (green), indicating that the lymph node is a false positive

*Step 3. Filtering of pseudolabels with anatomical structures:* False positive lymph nodes are filtered out based on the anatomy of the lungs and the mediastinum. Computed lymph nodes outside the mediastinum region and inside the mediastinum with significant overlap with mediastinal anatomical structures are excluded. The filtering is performed using segmentations of the chest structures computed with TotalSegmentator [4] (Fig. 2).

First, extra-mediastinal filtering uses the lung segmentation as the region of interest (ROI)—computed lymph nodes outside the ROI are excluded since mediastinal lymph nodes are, by definition, located between the lungs. Then, intramediastinal filtering removes computed lymph nodes that significantly overlap with mediastinal anatomical structures, as these often represent misclassified vessels or tissue with imaging characteristics similar to those of lymph nodes. The 17 relevant anatomical structures are: the trachea, bronchi, lungs, subclavian arteries, superior vena cava, pulmonary veins, common carotid arteries, branchiocephalic veins, branchiocephalic trunk, left atrial appendage, aorta, heart, esophagus, and pulmonary arteries. Significant overlap between a computed lymph node and a mediastinal structure is defined as at least 40% of voxels labeled in both. This threshold is set relatively high since lymph nodes are naturally adjacent to these anatomical structures, resulting in an acceptable overlap in the segmentations. This is particularly true for enlarged lymph nodes, where the boundary between the node and neighboring structures is difficult to establish accurately. A lower threshold could inappropriately exclude valid lymph nodes that are correctly detected but partially overlap with their anatomical neighbors.

*Step 4: Final model training:* A final 3D nnU-Net model with the same architecture and loss function as the ensembled models is trained with the resulting filtered pseudolabels using a standard training regime.

*Inference:* The resulting model is directly used for voxel-level mediastinum lymph classification on an entire CT scan without the ensembling, lungs structure segmentation, or filtering. Lymph node segmentations are obtained by computing 3D connected components and applying morphological operations to fill-in holes and remove small components ($\le$ 30 voxels, ~ 3 mm). SAL measurements are directly obtained from the segmentations.

## Results

*Datasets:* Three datasets were collected as follows: The private Hadassah dataset consists of scans of patients with enlarged lymph nodes undergoing follow-up examinations from two Hadassah University Medical Centers (Jerusalem, Israel). It includes chest ceCT scans acquired on Philips CT Brilliance iCT, Canon CT Aquilion Prime SP, and GE CT Optima 660 scanners. The public NIH dataset consists of ceCT scans from patients with enlarged mediastinal lymph nodes collected by the National Institutes of Health Clinical Center from various undisclosed clinical sites [28]. While scanner specifications are not detailed in [28], annotations, including manual lymph node segmentations, were provided by radiologists from this Center. The public LNQ2023 dataset consists of ceCT scans of oncology patients enrolled in clinical trials acquired between 2007 and 2020 from three U.S. academic medical centers (Massachusetts General Hospital, Dana Farber Cancer Institute, and Brigham and Women’s Hospital) via the Tumor Imaging Metrics Core (TIMC) [29]. According to [29], all annotators for the LNQ2023 dataset were trained radiologists or radiology domain experts with over 10 years of experience, with initial localizations performed by TIMC staff and US-board certified radiologists, and segmentations extended by project annotators. These scans were acquired on GE Healthcare Discovery CT750HD, GE Medical System BrightSpeed, Siemens SOMATOM Definition, Toshiba Aquilion, and Philips iCT scanners.

**Fig. 3:**
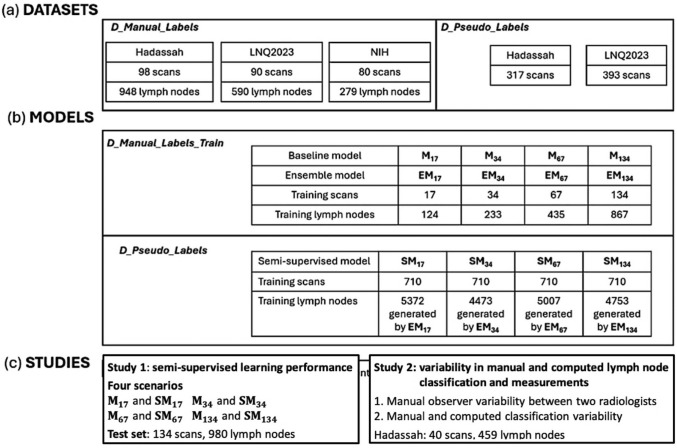
Overview of: **a** datasets of the manually labeled lymph nodes from three sources (Hadassah, LNQ2023, NIH) and the unlabeled data; **b** deep learning models trained with labeled (**M**_*i*_ and Ensemble, **EM**_*i*_) and unlabeled data (Semi-supervised, **SM**_*i*_) and the number of scans used for each. The index *i* = 17, 34, 67, 134 indicates the number of scans used to train the model; **c** two experimental studies, their models, and test sets

Four additional datasets were created (Fig. 3). Dataset ***D_Manual_Labels*** included 268 scans (98 Hadassah, 80 NIH, 90 LNQ2023). It was evenly split into training (***D_Manual_Labels_Train***) and test (***D_Manual_Labels_Test***) datasets of 134 scans each, with the same proportion of scans from each source. Dataset ***D_Pseudo_Labels*** consisted of 710 scans (317 Hadassah, 393 LNQ2023).

*Manual annotation:* Two coauthor senior radiologists (R1, R2) manually annotated a subset of the ***D_Manual_Labels*** scans using ITK-SNAP [30]. Prior to measuring, R1 marked each scan with a point indicating the locations of the mediastinal lymph nodes to be measured (Fig. S1, Supplemental Material). This ensured that both radiologists measured the same lymph nodes. Then, for these lymph nodes, SAL measurements and contour delineations in each CT axial slice were independently created by each radiologist. Finally, lymph nodes were classified into normal (SAL < 10 mm) and enlarged (SAL $\ge$ 10 mm) [11].

SAL measurements were obtained for 451 pre-selected lymph nodes in 40 scans of the Hadassah dataset. The reference slice, the short axis segment, and SAL were recorded. Contour delineations of 1,817 mediastinal lymph nodes were created on the pre-selected lymph nodes for 268 scans in ***D_Manual_Labels***. Of those, 1,073 were normal and 744 were enlarged lymph nodes. R1 manually segmented the lymph nodes in the 98 scans of the Hadassah dataset, yielding 720 normal (mean 4.3 per scan) and 228 enlarged lymph nodes (mean 2.3 per scan). For the 90 scans in the LNQ2023 dataset, the segmentations provided with the dataset were used. They consist of 335 normal (mean 3.7 per scan) and 255 enlarged lymph nodes (mean 2.8 per scan). For the 80 scans in the NIH dataset, the lymph node segmentations provided with the dataset were used. They consist of 18 normal (mean 0.2 per scan) and 261 enlarged lymph nodes (3.2 per scan).

*Deep learning models:* Three types of deep learning models for lymph node detection and segmentation were created and evaluated for four training set sizes, ***n*** = 17, 34, 67, 134 (Fig. 3): (1) ***Baseline models:* M**_**17**_, **M**_**34**_, **M**_**67**_, **M**_**134**_ are 3D nnU-Net models trained on ***n*** manually annotated scans. (2) ***Ensemble models*: EM**_**17**_, **EM**_**34**_, **EM**_**67**_, **EM**_**134**_ are ensembles of 24 3D nnU-Net models trained as described in Sect. "Method". ” They were used to compute pseudolabels on ***D_Pseudo_Labels***. (3) **Semi-supervised models: SM**_**17**_, **SM**_**34**_, **SM**_**67**_, **SM**_**134**_ are 3D nnU-Net models trained on filtered pseudolabels generated by their corresponding **EM** models. All models** M**_***i***_ and **SM**_**i**_ were evaluated on ***D_Manual_Labels_Test***, consisting of 134 scans with 614 normal and 366 enlarged lymph nodes.

We used the TotalSegmentator model to obtain organ segmentations for 710 unlabeled scans. Since there is no ground truth available, we evaluated the results visually. Moreover, even if the results contain errors, they are only used for filtering. Our qualitative evaluation of the results of the TotalSegmentator indicated good performance on our datasets.

*Evaluation metrics:* Agreement between observers and between observers and computed measures was quantified with a confusion matrix for normal and enlarged lymph node classes. Automatic lymph node detection was evaluated with standard precision and recall. Lymph node segmentation was evaluated with the Dice score and ASSD for normal and enlarged lymph nodes. SAL measurements were evaluated with the absolute difference between SAL measurements (${\Delta }$*SAL*). The axial slice difference between measurement locations, ***slice difference*** (Δ*Slice*), and the angular difference between measurement axes’ orientations, ***angle difference*** (Δ*Angle*) were also computed. These metrics were computed for the manual computed measurements for each scan for a set of scans with mean (std) Dice score, ASSD, and mean and maximum SAL difference.

Formally, the SAL of a connected component in the binary voxel classification mask is defined as follows: Let $C$ be a set of voxels of a 2D-connected component, let $\partial C$ = $contour\left( C \right)$ be the contour, let $d\left( {p_{a} , p_{b} } \right)$ be the Euclidean distance between two points $p_{a}$, $p_{b}$. Let $p_{L1}$, $p_{L2}$ be two extremal points on the boundary, defined by:

$$ (p_{L1} ,p_{L2} ) = \mathop {{\mathrm{argmax}}}\limits_{{\left( {p_{a} , p_{b} } \right) \in \partial C \times \partial C}} d\left( {p_{a} , p_{b} } \right) $$

The normalized long axis vector $\vec{u}_{LA}$ is:

$$ \vec{u}_{LA} = \frac{{\left( {p_{L1} - { }p_{L2} } \right){ }}}{{d\left( {p_{L1} ,{ }p_{L2} } \right)}} $$

Then, the short axis of $C$ is:

$$SAL_{2D} \left( C \right) $$ = $$\mathop {\max }\limits_{{\left( {p_{a} , p_{b} } \right) \in \partial C \times \partial C}} d\left( {p_{a} , p_{b} } \right)$$ such that $$\left( {p_{a} - { }p_{b} } \right)$$. $$\vec{u}_{LA}$$ = 0.

*Experimental studies:* We conducted two experimental studies as follows: Study 1 evaluated our method for four model training scenarios and compared them with the supervised learning method. Study 2 evaluated the variability in manual and computed lymph node classification and short axis length measurements performed by two radiologists and measurements computed from lymph node segmentations.

**Fig. 4:**
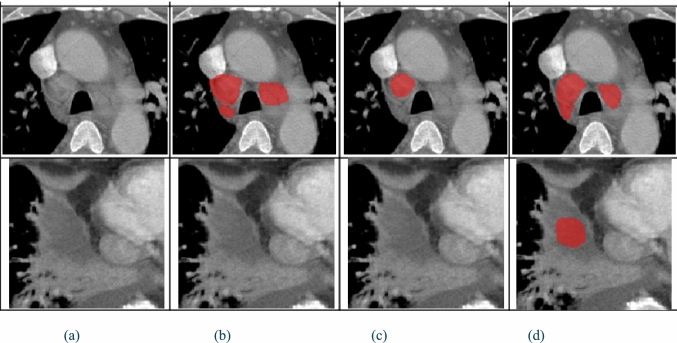
Study 1. Two examples of lymph node segmentation results generated by the baseline model M_17_ trained on 17 annotated scans and its semi-supervised counterpart SM_17_. Columns show **a** the original CT slice, **b** the ground truth (GT), **c** the M_17_ computed segmentation, and **d** the SM_17_ computed segmentation. The top row shows an example of the improved recall of SM_17_, which detected both lymph nodes while M_17_ missed one. The bottom row shows an example of the reduced precision of SM_17_, with a false positive not detected by M_17_

Study 1: Semi-supervised learning performance The performance of the final 3D nnU-Net models was quantified for four scenarios with increasing amounts of training data from ***D_Manual_Labels_Train***. Each scenario evaluates two model variants: (1) Reference model ${\mathbf{M}}_{{\mathbf{i}}}$ was trained on ***i*** manually annotated scans; (2) Semi-supervised model ${\mathbf{SM}}_{{\mathbf{i}}}$ was trained on pseudolabels generated for ***D_Pseudo_Labels*** using ensemble model ${ }{\mathbf{EM}}_{{\mathbf{i}}}$. The scenarios used training sets of 17 scans (68 normal and 56 enlarged lymph nodes), 34 scans (132 normal and 101 enlarged lymph nodes), 67 scans (253 normal and 182 enlarged lymph nodes), and 134 scans (493 normal and 374 enlarged lymph nodes). Prior to training the ${\mathbf{SM}}_{{\mathbf{i}}}$ models, the ensemble models ${\mathbf{EM}}_{{\boldsymbol{i}}}$ generated pseudolabels on the ***D_Pseudo_Labels*** dataset. After filtering, **EM**_**17**_ classified 3,711 normal and 1,661 enlarged lymph nodes, **EM**_**34**_ classified 3,003 normal and 1,470 enlarged lymph nodes, **EM**_**67**_ classified 3,510 normal and 1,497 enlarged lymph nodes, and **EM**_**134**_ classified 3,268 normal and 1,485 enlarged lymph nodes. Figure 4 shows two examples of the results.

**Fig. 5:**
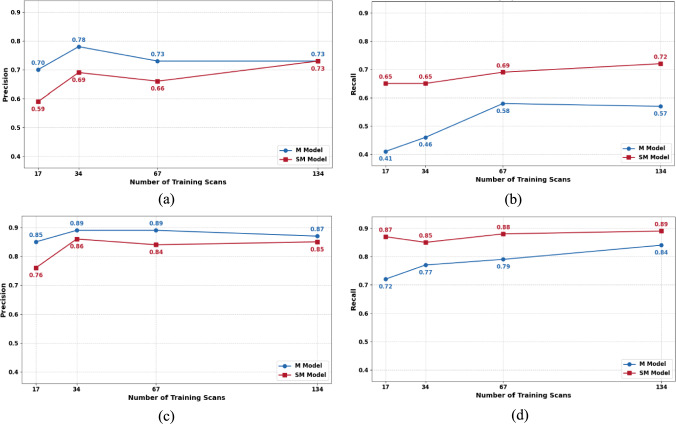
Results of Study 1. Performance of the supervised (**M**) and semi-supervised (**SM**) models for lymph node detection: **a** precision and **b** recall for normal lymph nodes for training sets of sizes 17, 34, 67, 134; **c** precision and **d** recall for enlarged lymph nodes for training sets of sizes 17, 34, 67, 134

Table 1 lists the results. Figure 5 shows the graphs. For enlarged lymph nodes, the **SM** models consistently achieved higher detection recall than their **M** counterparts, with improvements of 15% (0.87 vs. 0.72) for **SM**_**17**_, 8% (0.85 vs. 0.77) for **SM**_**34**_, 9% (0.88 vs. 0.79) for **SM**_**67**_, and 5% (0.89 vs. 0.84) for **SM**_**134**_, with a small decrease of 2–9% in precision for all. Segmentation quality was similar for corresponding **M** and **SM** models, with Dice scores within 2–5%. The **SM** models, however, showed modest improvements in *ASSD*, particularly **SM**_**67**_ with a 15% reduction compared to **M**_**67**_ (4.37 mm vs. 5.15 mm). For normal lymph nodes, the recall improvements were even more pronounced: 24% (0.65 vs. 0.41) for **SM**_**17**_, 19% (0.65 vs. 0.46) for **SM**_**34**_, 11% (0.69 vs. 0.58) for **SM**_**67**_, and 15% (0.72 vs. 0.57) for **SM**_**134**_, with a larger decrease of 5–11% in precision compared to enlarged lymph nodes. For segmentation, there were minimal differences between **M** and **SM** models, with Dice score differences of 3–7%.

**Table 1:** Results of Study 1

Scenario	Detection		Segmentation			
	Precision	Recall	Dice	ASSD (mm)	Mean ${\Delta }$*SAL* (mm)	Max $\Delta SAL$ (mm)
M_17_	0.70 (0.38)	0.41 (0.35)	0.66 (0.27)	2.13 (1.79)	1.59 (0.83)	2.21 (1.21)
SM_17_	0.59 (0.30)	0.65 (0.31)	0.59 (0.26)	2.63 (3.30)	1.59 (1.01)	2.80 (2.12)
M_34_	**0.78 (0.31)**	**0.46 (0.35)**	**0.67 (0.27)**	**2.43 (3.49)**	**1.59 (1.00)**	**2.42 (1.76)**
SM_34_	**0.69 (0.32)**	**0.65 (0.32)**	**0.59 (0.26)**	**2.53 (3.16)**	**1.74 (0.98)**	**2.93 (2.47)**
M_67_	0.73 (0.33)	0.58 (0.34)	0.66 (0.24)	1.87 (1.72)	1.57 (1.16)	2.74 (2.97)
SM_67_	0.66 (0.31)	0.69 (0.29)	0.60 (0.25)	2.31 (3.27)	1.59 (0.82)	2.74 (1.77
M_134_	0.73 (0.32)	0.57 (0.33)	0.65 (0.24)	2.02 (2.34)	1.73 (1.34)	3.20 (3.35)
SM_134_	0.73 (0.29)	0.72 (0.28)	0.62 (0.25)	2.31 (3.21)	1.65 (0.92)	3.03 (1.91)

Performance of lymph node detection and segmentation for four training scenarios on test set ***D_Manual_Labels_Test*** (134 scans, 614 normal lymph nodes) for four training set sizes, *i* = 17, 34, 67, 134 and four baseline **(M**_*i*_) and pseudolabels (**SM**_*i*_) models. Listed are the mean (std) lymph node detection precision and recall, the mean (std) segmentation Dice coefficient and ASSD in mm, the mean and maximum SAL differences in mm between the computed and the manual measurements. Highlighted is the best performing model (bold)

The **SM** models consistently achieved better mean SAL measurements compared to their **M** counterparts, with notable Δ*SAL* improvements of 0.52 mm for **SM**_**17**_ (5.16 mm vs. 5.68 mm) and 1.45 mm for **SM**_**67**_ (4.01 mm vs. 5.46 mm) for enlarged lymph nodes. Maximum Δ*SAL* values were also better for **SM** models, particularly for **SM**_**67**_, with a 2.47 mm improvement (6.19 mm vs. 8.66 mm). For normal lymph nodes, the mean Δ*SAL* remained similar, 0.15 mm.

The best performing semi-supervised model, **SM**_**134**_, achieved mean SAL differences (± std) of 1.65 ± 0.92 mm for normal lymph nodes and 4.25 ± 4.98 mm for enlarged lymph nodes, within the observer variability. Models trained with substantially fewer annotated scans, e.g. **SM**_**17**_ using 1/8th the data of **M**_**134**_, achieved comparable or superior recall for enlarged lymph nodes.

Table 2 lists results of the pseudolabels filtering. The table lists the number of lymph nodes computed by the four ensemble models **EM**_*i*_ with training set sizes, *i* = 17, 34, 67, and 134 before and after filtering. The filtering reduces by 8.4–12.5% (448–677) the number of lymph nodes that are used to train the pseudolabels models **SM**_*i*_. Note that the reduction is significant, as all the lymph nodes that are filtered out are false positives and should not be used for model training.

**Table 2:** Results of Study 1

Ensemble Model	LNs before filtering	LNs removed by filtering	Filtered LNs used for SM training	LNs reduction (%)
EM_17_	5901	529	5372	9.0
EM_34_	4921	448	4473	9.1
EM_67_	5465	458	5007	8.4
EM_134_	5430	677	4753	12.5

Performance of the pseudolabels filtering for four ensemble models (**EM**_*i*_) with training set sizes, *i* = 17, 34, 67, 134. Listed are the number of lymph nodes (LNs) detected with pseudolabels before filtering, the number of LNs removed by filtering, the number of LNs after filtering used to train the pseudolabels models (**SM**_*i*_) and the reduction of False Positive LNs in %

Study 2: Variability in manual and computed lymph node classification and measurements.

*Manual observers*’* measurements:* Radiologists R1 and R2 independently measured the SAL of 459 lymph nodes in 40 CT scans from ***D_Manual_Labels***. R1 marked the lymph nodes to be measured 4 weeks before the measurements were performed. Both radiologists then measured the marked lymph nodes (Fig. S1, Supplemental Material).

Table 3 lists the results. The radiologists agreed on the classification of 94% (423) of the lymph nodes, with 82% (371) classified as normal and 12% (52) as enlarged by both radiologists. They disagreed on 6% (28) lymph nodes, with 4% (18) classified as normal by R1 and enlarged by R2, and 10 (2%) classified as enlarged by R1 and normal by R2. These results show a high classification agreement.

**Table 3:** Results of Study 2

	Category	# of lymph nodes (%)	R1 SAL (mm)	R2 SAL (mm)	Δ*SAL* (mm)	${\Delta }$ *Slice*	${\Delta }$ *Angle* (degrees)
Agreement	R1: Normal R2: Normal	371 (82%)	5.4 (1.7)	6.0 (1.8)	1.1 (0.9)	0.8 (2.2)	31 (24)
	R1: Enlarged R2: Enlarged	52 (12%)	14.7 (4.5)	14.7 (4.3)	1.5 (1.6)	1.1 (1.4)	23 (20)
Disagreement	R1: Normal R2: Enlarged	18 (4%)	8.4 (1.3)	12.3 (3.1)	3.9 (3.9)	1.6 (2.2)	34 (26)
	R1: Enlarged R2: Normal	10 (2%)	11.2 (1.2)	8.3 (1.1)	2.9 (1.9)	1.3 (2.1)	44 (23)

Lymph node classification as normal and enlarged between two radiologists, R1 and R2 and their measurements of the short axis length (SAL) of mediastinal lymph nodes. The results are categorized into two agreement cases where both radiologists classified lymph nodes as normal (< 10 mm) or enlarged ($\ge$ 10 mm) and two disagreement cases where radiologists differed. Measurements include absolute difference between SAL measurements (${{\boldsymbol{\Delta}}}$***SAL***), difference in measurement slice location (${{\boldsymbol{\Delta}}}$***Slice***), and the difference in measurement angle (${{\boldsymbol{\Delta}}}$***Angle***).

Detailed measurement analysis: for lymph nodes classified as normal by both radiologists, the mean SAL was 5.7 ± 1.8 mm and the mean SAL absolute difference was 1.1 ± 0.9 mm. For enlarged lymph nodes, the mean SAL was 14.7 mm and the mean SAL absolute difference was 1.5 ± 1.6 mm. For cases where the classification differed, the SAL measurement differences were 3.9 ± 3.9 mm when R1 classified as normal and R2 as enlarged, and 2.9 ± 1.9 mm in the opposite case. Measurement variability was associated with differences in both slice selection (mean difference 0.8–1.6 slices) and angle of measurement (mean difference 23°–44°).

*Manual and computed measurements:* The agreement between manual measurements and radiologists R1 and R2 measurements computed from manual lymph node segmentations was quantified for the same lymph nodes as before.

Table 4 lists the results. Manual R1 and computed measurements agreed on 93% (421) of the lymph nodes, with 82% (368) classified as normal and 12% (53) as enlarged. Disagreement was on 5% (21) lymph nodes, with 2% (9) classified as normal by R1 and enlarged by computation, and 2% (10) classified as enlarged by R1 and normal by computation. Similarly, manual R2 and computed measurements agreed on 92% (415) of the lymph nodes, with 80% (361) classified as normal and 12% (54) as enlarged. Disagreement was on 8% (36) lymph nodes, with 4% (20) classified as normal by R2 and enlarged by computation, and (4%) classified as enlarged by R2 and normal by computation. This indicates strong agreement between the manual and computed measurements, nearly identical to the observer variability.

**Table 4:** Results of Study 2

R1		Computed		R2		Computed	
		Normal	Enlarged			Normal	Enlarged
Manual	Normal	368 (82%)	21 (5%)	Manual	Normal	361 (80%)	20 (4%)
	Enlarged	9 (2%)	53 (12%)		Enlarged	16 (4%)	54 (12%)

Confusion matrices of lymph node classification as normal and enlarged: (a) R1 and computed measurements and (b) R2 and computed measurements

## Discussion

Our semisupervised learning method for automatic detection and segmentation of mediastinal lymph nodes in ceCT scans combines a small set of expert-annotated scans with a large set of unannotated scans to improve lymph node detection and segmentation performance while reducing annotation burden. The final model is a standard 3D nn-U-Net trained in a fully supervised mode. It requires from one-fourth to one-eighth less annotated data (tens vs. hundreds of scans) to achieve a performance that is similar to a fully supervised method.

Our studies demonstrate that the final models achieved substantial improvements in recall over their supervised counterparts with slightly lower precision levels. With only 17 annotated scans and 710 unlabeled scans, the semi-supervised model increased the recall by 24% and 15% for normal and enlarged lymph nodes to 0.65 and 0.87, with a 9% and 5% decrease in precision and similar Dice scores. The recall improvements persisted when more annotated training data became available: with 134 annotated scans, the recall was 5% higher (0.89 vs. 0.84). This recall/precision trade-off may be advantageous since reviewing misidentified lymph nodes typically requires less effort than searching for missed ones. Also, this result suggests that the pseudolabels generated by the ensemble models provide valuable complementary information beyond what is captured by the manual annotations. The study sheds light on the precision-recall trade-off, which is task- and structure-specific for different data training regimes.

The observer variability results highlight the inherent subjectivity in manual lymph node measurements. The classification agreement between radiologists (94%) and between manual and computed measurements (92–93%) demonstrates the reliability of automatic SAL lymph node measurements and underscores the potential value of automated methods in providing accurate and reproducible measurements. The mean SAL differences of the best performing model were 1.65 ± 0.92 mm and 4.25 ± 4.98 mm for normal and enlarged lymph nodes. The larger SAL measurement differences of 2.9–3.9 mm that were observed when the radiologists disagreed on the lymph node classification emphasize the need for consistent measurement techniques for lymph nodes with SALs close to 10 mm.

Note that the resulting model for online inference on new scans is computationally efficient (a few seconds), as it does not require the use of the TotalSegmentator. Training is performed offline, with a standard computational cost of several hours, including the false positive filtering using the results of the segmentation of the chest structures in the CT scans with the TotalSegmentator model [6].

*Limitations:* First, the quality of the CT scans and of the lymph node segmentations in the two public datasets was not validated; annotations of normal lymph nodes were not available for the NIH dataset. Consequently, we could not determine if there is a bias in the dataset. Second, the observer variability study included radiologists from the same institution and only 40 scans, which may not fully capture the range of variability across different clinical settings and external observers. Third, the effectiveness of anatomical filtering depends on the anatomical structure segmentation results provided by the TotalSegmentator. When these segmentation results are inaccurate, they may affect the performance of the final model.

## Conclusion

Label-efficient deep learning methods for automated detection, segmentation, and measurement of anatomical structures in volumetric imaging are essential for the scalable development of computer-assisted diagnostic tools for hundreds of small anatomical structures and pathologies. We have presented a semi-supervised framework that generates anatomically filtered pseudolabels to augment limited manual annotations, specifically applied to the detection of enlarged mediastinal lymph nodes in chest ceCT. Our results show that performance comparable to fully supervised models can be achieved using only one-fourth to one-eighth of the manual annotations required for lymph node labeling. This highlights the potential of pseudolabeling with anatomical filtering as a practical approach to reduce the manual annotation burden, thereby facilitating the development and clinical deployment of AI-driven radiology applications.

## Supplementary Information

Below is the link to the electronic supplementary material.

Supplementary file1 (DOCX 246 KB)
