# Supplementary material for: Annotation-efficient deep learning detection and measurement of mediastinal lymph nodes in CT
Source: Int J Comput Assist Radiol Surg. 2025 Sep 13;21(1):103–13. doi: 10.1007/s11548-025-03513-y (PMC12929302; doi:10.1007/s11548-025-03513-y)
Supplement: Supplementary file 1 — Supplementary file1 (DOCX 246 KB) [file 11548_2025_3513_MOESM1_ESM.docx]

***Supplemental Material***

**Annotation-Efficient Deep Learning Detection and Measurement of Mediastinal Lymph Nodes in CT**

Alon Olesinski^1^ MSc, Richard Lederman^2^ MD, Yusef Azraq^2^ MD, Jacob Sosna^2^ MD, Leo Joskowicz^1^* PhD

1. School of Computer Science and Engineering, The Hebrew University of Jerusalem, Israel.

2. Dept. of Radiology, Hadassah University Medical Center, Jerusalem, Israel.

**3. Experimental results**

***Study 2: Observer variability in manual lymph node measurements***


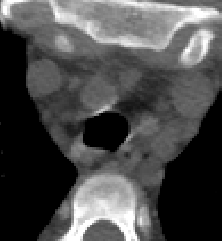

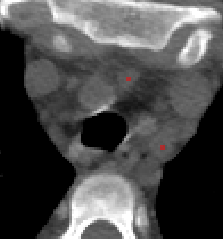

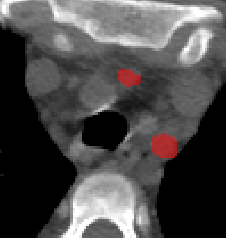

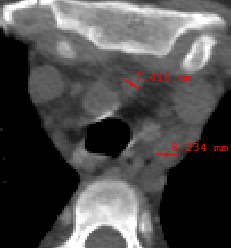


(a) (b) (c) (d)

**Fig. S1. Study 2. Illustration of manual annotations**. Two annotations of lymph nodes (red): (a) detail of the reference CT scan slice; (b) pre-selection markings; (c) contour delineation; (d) short axis measurements.


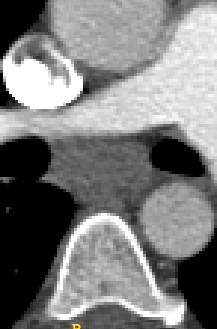

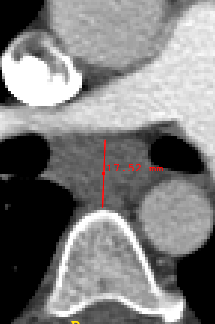

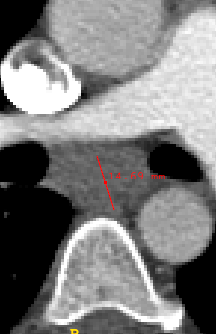


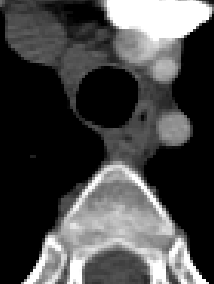

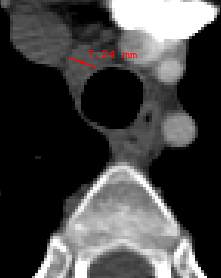

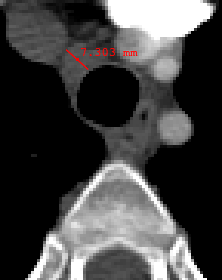


(a) (b) (c)

**Fig. S2. Illustration of Study 2.** Two examples of inter-observer variability in lymph node short axis measurements. Each row shows the detail of the reference axial slice (left), R1's measurement (middle), and R2's measurement (right). The top row shows high discrepancy between both radiologists in measuring an enlarged lymph node due to a different angle selection and irregular lymph node shape, which usually leads to higher inter-observer variability. The bottom row shows a significant measurement agreement in a normal lymph node.
